# Tau Secretion: Good and Bad for Neurons

**DOI:** 10.3389/fnins.2019.00649

**Published:** 2019-06-26

**Authors:** Camille Pernègre, Antoine Duquette, Nicole Leclerc

**Affiliations:** ^1^Research Centre of the University of Montreal Hospital (CRCHUM), Montréal, QC, Canada; ^2^Département de Neurosciences, Faculty of Medicine, Université de Montréal, Montréal, QC, Canada

**Keywords:** tau protein, secretion, tauopathies, extracellular tau, propagation

## Abstract

In Alzheimer’s disease (AD), neurofibrillary tangles (NFTs), lesions composed of hyperphosphorylated and aggregated tau, spread from the transentorhinal cortex to the hippocampal formation and neocortex. Growing evidence indicates that tau pathology propagates *trans-*synaptically, implying that pathological tau released by pre-synaptic neurons is taken up by post-synaptic neurons where it accumulates and aggregates. Observations such as the presence of tau in the cerebrospinal fluid (CSF) from control individuals and in the CSF of transgenic mice overexpressing human tau before the detection of neuronal death indicate that tau can be secreted by neurons. The increase of tau in the CSF in pathological conditions such as AD suggests that tau secretion is enhanced and/or other secretory pathways take place when neuronal function is compromised. In physiological conditions, extracellular tau could exert beneficial effects as observed for other cytosolic proteins also released in the extracellular space. In such a case, blocking tau secretion could have negative effects on neurons unless the mechanism of tau secretion are different in physiological and pathological conditions allowing the prevention of pathological tau secretion without affecting the secretion of physiological tau. Furthermore, distinct extracellular tau species could be secreted in physiological and pathological conditions, species having the capacity to induce tau pathology being only secreted in the latter condition. In the present review, we will focus on the mechanisms and function of tau secretion in both physiological and pathological conditions and how this information can help to elaborate an efficient therapeutic strategy to prevent tau pathology and its propagation.

## Observations Supporting Tau Secretion by Neurons

Tau protein, an axonal microtubule-associated protein, accumulates both intracellularly and extracellularly in AD. Extracellular tau is presently a promising therapeutic target to block the propagation of tau pathology in the brain of AD patients. Based on this, the elucidation of tau routes to the extracellular space could serve to elaborate therapies for prevention of the tau pathology spreading and the cognitive deficits linked to it. The presence of tau in CSF and the medium of neuronal cultures suggested that neurons can secrete it. However, its accumulation in the CSF during the progression of AD was believed to correlate with neuronal cell death (Hampel et al., 2010). The interneuronal transfer of human tau overexpressed in anterior bulbar neurons of the lamprey was the first observation indicating that tau could be secreted by neurons *in vivo* (Kim et al., 2010). More recently, tau release by neurons *in vivo* was demonstrated by its detection in the interstitial fluid in the absence of neurodegeneration using microdialysis in tau transgenic mouse brain (Yamada et al., 2011). Neuronal activity was shown to increase tau release by neurons both *in vitro* and *in vivo* (Pooler et al., 2013; Yamada et al., 2014). In particular, neuronal hyperexcitability observed at early stages of both sporadic and familial AD and in AD mouse models was shown to increase tau release (Busche et al., 2008, 2012; Palop and Mucke, 2009; Bakker et al., 2012; Pooler et al., 2013; Wu et al., 2016). Furthermore, we reported that the induction of autophagy and/or lysosomal dysfunction increased the release of tau by primary cortical neurons (Mohamed et al., 2014). Intriguingly, we recently reported that tau secretion induced by neuronal hyperactivity was associated with Golgi fragmentation indicating that Golgi membranes may play a role in tau secretion (Mohamed et al., 2017). Consistent with this, blocking the fragmentation of the Golgi upon the induction of hyperactivity reduced tau secretion. Lastly, the pattern of tau forms found in the medium upon cell damage differs from that resulting from active secretion (Plouffe et al., 2012; Kanmert et al., 2015). All the above observations demonstrate that neurons can secrete tau and that changes in its levels of secretion are linked to pathological conditions.

## Mechanisms Involved in Membrane Free-Tau and Vesicle/Exosome-Tau Secretion

**FIGURE 1:**
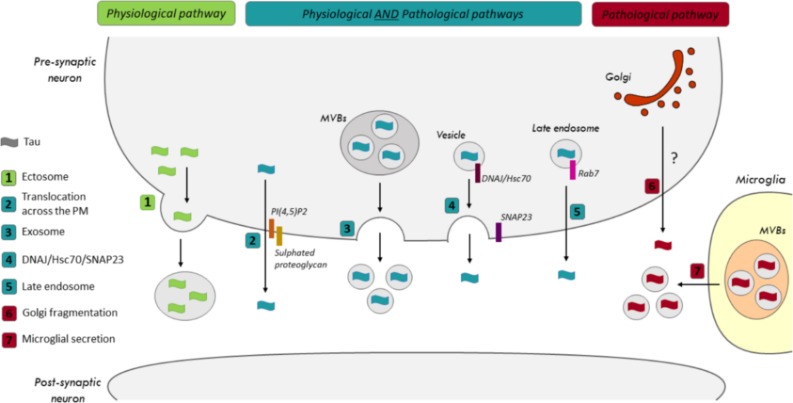
Secretory pathways of tau in physiological and pathological conditions. (1) The release of tau by ectosomes was only reported in physiological conditions. In both physiological and pathological conditions, tau could be released by (2) its translocation across the plasma membrane (PM), (3) exosomes, (4) the SNARE, SNAP-23, and the co-chaperone DNAJ/Hsc70 and (5) Rab7A, a Rab GTPase associated with late endosomes (LEs). (6) A correlation was noted between the fragmentation of the Golgi and an increase of tau secretion in pathological conditions. However, the pathway is not characterized yet. (7) Microglia were shown to release exosomes containing pathological tau in a tauopathy mouse model.

Two pools of extracellular tau are detected: a small pool found in vesicles and a large membrane-free pool (Saman et al., 2012; Pooler et al., 2013; Dujardin et al., 2014; Mohamed et al., 2014). Several groups, including ours, have demonstrated that neurons release tau by unconventional secretory pathways, rather than by the conventional endoplasmic reticulum (ER)-Golgi pathway, in both physiological and pathological conditions (ex. starvation, lysosomal dysfunction and hyperactivity) (Pooler et al., 2013; Mohamed et al., 2014, 2017). Both non-vesicular (free-tau) and vesicular (vesicle/exosome-tau) mechanisms were shown to contribute to tau secretion (Figure 1).

### Translocation Across the Plasma Membrane

Two recent studies reported that both endogenous tau and overexpressed human tau can be secreted by a non-vesicular mechanism involving its direct translocation across the plasma membrane (PM) (Katsinelos et al., 2018; Merezhko et al., 2018). This mechanism was previously described for the fibroblast growth factor 2 (FGF2) (Steringer et al., 2017). Unconventional secretion of FGF2 occurs through a mechanism of self-sustained membrane translocation with FGF2 forming its own membrane translocation intermediates by reversible oligomerization, membrane insertion and disassembly in the extracellular space. As noted for FGF2, the interaction of tau with PI(4,5)P_2_ and sulphated proteoglycans mediates its translocation (Katsinelos et al., 2018; Merezhko et al., 2018). PI(4,5)P_2_ are involved in the binding of tau at the inner surface of the PM and sulphated proteoglycans found at the cell surface facilitate the export process. Auxiliary factors such as ATP1A1 and Tec kinase located at the PM are involved in FGF2 secretion (Ebert et al., 2010; Zacherl et al., 2015). Such factors remain to be identified for tau. As noted for FGF2, the formation of oligomer intermediates was shown to be necessary for tau secretion by this mechanism (Merezhko et al., 2018). The properties of the PM also modulate tau secretion. For example, the reduction of specific lipid species such as cholesterol and sphingomyelin impaired tau secretion (Merezhko et al., 2018). Interestingly, the secretion of phosphorylation-mimicking tau mutant through this mechanism was significantly more important than that of wild-type tau and a non-phosphorylatable tau mutant (Merezhko et al., 2018).

### Membranous Organelles-Based Tau Secretion

The contribution of membranous organelles to tau secretion was revealed by demonstrating that SNAREs and Rabs, two classes of proteins implicated in membrane trafficking are involved in tau secretion. In a recent study, it was observed that the suppression of SNAP-23, a SNARE protein implicated in neurotransmitter exocytosis, resulted in a significant reduction of tau secretion (Fontaine et al., 2016). The co-chaperone DNAJ/Hsc70 was shown to be necessary for tau secretion through this pathway. However, in this study, the membranous compartment involved in tau secretion was not characterized. This secretory pathway was shown to take place in both physiological and pathological conditions and to be used by wild-type and mutated human tau (P301L and R406W) (Fontaine et al., 2016).

We recently investigated tau secretory pathways (Plouffe et al., 2012; Mohamed et al., 2013, 2014, 2017; Rodriguez et al., 2017) and showed that late endosomes (LEs) are involved in the unconventional secretion of membrane-free tau (Rodriguez et al., 2017). Our data revealed that LEs could secrete membrane-free tau via Rab7A, a GTPase associated with these endosomes and involved in their trafficking (Rodriguez et al., 2017). Tau partially co-localized with Rab7A-positive structures and its secretion was significantly reduced by Rab7A depletion (Rodriguez et al., 2017). Interestingly, LEs were recently shown to be involved in the secretion of the huntingtin mutant (mHtt) linked to Huntington’s disease (Trajkovic et al., 2017). At early stages of AD when tau intracellular accumulation occurs, Rab7A is upregulated and there is an accumulation of endosomal structures (Nixon, 2005; Ginsberg et al., 2010a, b, 2011; Tiernan et al., 2016). Based on our above data, all these changes could favor tau secretion and its accumulation in the CSF of AD patients. In yeast, endosomes contribute to the formation of a compartment for unconventional protein secretion (CUPS), which arises following starvation (Cruz-Garcia et al., 2014; Curwin et al., 2016). We have reported that tau secretion is increased upon starvation (Mohamed et al., 2014). Thus, one can speculate that the accumulation of endosomes noted in AD may contribute to the production of CUPS yielding increased tau secretion.

We also reported that Rab1A was involved in tau secretion (Mohamed et al., 2017). Rab1A is associated with the membranes of the Golgi apparatus and its suppression induces their fragmentation (Shorter and Warren, 2002; Coune et al., 2011). In AD and tau transgenic mice, a fragmentation of the Golgi membranes was observed (Dal Canto, 1996; Stieber et al., 1996; Liazoghli et al., 2005; Anton-Fernandez et al., 2016). We showed that inducing Golgi fragmentation by suppression of Rab1A increased tau secretion (Mohamed et al., 2017).

### Exosomes/Vesicles

Exosomes and ectosomes are the two main types of extracellular vesicles involved in the secretion of cytosolic proteins (Kalra et al., 2012). In the case of exosomes, cytosolic proteins are captured from the cytoplasm during the formation of internal endosomal vesicles resulting in the generation of multi-vesicular bodies (MVBs) (Hessvik and Llorente, 2018). Upon the fusion of MVBs with the PM, exosomes are released in the extracellular space. Ectosomes are larger than exosomes and result from the shedding of the PM (Muralidharan-Chari et al., 2010). Both exosomes and ectosomes were shown to be involved in tau secretion (Saman et al., 2012; Simon et al., 2012; Dujardin et al., 2014; Asai et al., 2015; Kanmert et al., 2015; Polanco et al., 2016; Wang et al., 2017; Guix et al., 2018). However, only a small pool of tau was found in these structures (1 to 10% of extracellular tau) (Kanmert et al., 2015). This mechanism was observed for endogenous tau and overexpressed human tau (Table 1). Vesicles containing tau leaving from anterior bulbar neurons in lamprey was the first observation indicating that tau could be released by exosomes/ectosomes (Lee et al., 2012). Several cell lines and primary neurons were reported to release tau by exosomes (Table 1). In pathological conditions, microglia were also shown to release tau by exosomes (Asai et al., 2015). In one study, tau was found in ectosomes (Dujardin et al., 2014). In this study, the authors observed the secretion of tau by ectosomes in physiological conditions whereas tau secretion by exosomes was observed in pathological conditions.

**TABLE 1:** Studies on tau secretory pathways.

**Tau isoforms and mutants**		**Cell types**		***In vivo***	**Secretion of tau**	**Characterization of secreted tau**	**Mode of secretion**		**References**
**Endogenous tau**	**Overexpressed tau**	**Cell lines**	**Primary neurons**				**Free**	**Vesicles**	
mouse	WT, P301L and R406W	HEK293T M17	Mouse cortical neurons. Organotypic brain slice from WT mice		WT = P301L = R406W		SNAP23 DNAJ/Hsc70		Fontaine et al.,2016
Rat	0N4R	HeLa	Rat cortical neurons			Hypophosphorylated and cleaved at the C-terminal	Late endosomes/Rab7A		Rodriguez et al.,2017
Rat	0N4R	HeLa	Rat cortical neurons			Hypophosphorylated and cleaved at the C-terminal	Golgi/Rab1A		Mohamed et al.,2017
Mouse	0N4R E14 (hyperphosphorylated) AP (mutant non-phosphorylable	SH-SY5Y	Mouse hippocampal neurons		E14 > WT and AP		Translocation across the PM		Katsinelos et al.,2018
Rat	0N4R	N2A	Rat cortical neurons			Dimers/trimers or tetramers β-sheet aggregates	Translocation across the PM		Merezhko et al.,2018
	pSGtau	COS-7 HEK293				FL-tau and truncated tau		Exosomes	Simon et al.,2012
	0N4R	M1C				C-terminal truncated tau. Phosphorylated at T181, S202/T205, T212/S214 and T231/S235		Exosomes	Saman et al.,2012
Rat	1N4R	NIE-115	Rat cortical neurons			FL-tau and N- and C-terminal truncated forms	X	Ectosomes and exosomes	Dujardin et al.,2014
	2N4R		microglia					Exosomes	Asai et al.,2015
	P301S (1N4R)			PS19 mice		Phosphorylated at S396/S404 oligomers		Exosomes	
Human		N2A	Human iPSC-derived cortical neurons			Non-aggregated	X	Exosomes	Kanmert et al.,2015
	WT and P301L			rTG4510 mice and WT mice	P301L > WT	Phosphorylated at T181, S262 and S422		Exosomes	Polanco et al.,2016
Rat	2N4R Δ280 (Tau RDΔk)	N2A	Rat cortical neurons			FL-tau. Low phosphorylation. Insoluble and aggregated		Exosomes	Wang et al.,2017
Human			Human iPSC-derived cortical neurons			FL-tau, N-terminal, and mid-region fragments	X	Exosomes	Guix et al.,2018

## Extracellular Tau Forms: Secreted Tau Vs. CSF-Tau

Although several studies indicate that tau found in CSF most likely results from its secretion by neurons, this remains to be experimentally demonstrated. Observations performed in lamprey where human tau was overexpressed in anterior bulbar neurons indicate that tau released by neurons could reach the CSF by periventricular and perimeningeal routes (Le et al., 2012). The fact that CSF-tau and secreted tau share similar post-translational modifications (PTMs) such as cleavage, phosphorylation and aggregation indicates that CSF-tau corresponds to secreted tau. In CSF obtained from control individuals and AD patients, both full-length tau (FL-tau) and truncated tau were detected (Johnson et al., 1997; Meredith et al., 2013; Wagshal et al., 2015; Guix et al., 2018). In all studies, the major form of tau present in CSF was cleaved at the C-terminal. In the medium from neuronal cultures, the amount of FL-tau varies from one study to another. In some studies, FL-tau was the main form of tau whereas in other studies it was the minor one (less than 1% of total secreted tau) (Plouffe et al., 2012; Pooler et al., 2013; Mohamed et al., 2014, 2017; Bright et al., 2015; Kanmert et al., 2015; Rodriguez et al., 2017). In transgenic mice, extracellular FL-tau was the main form of tau detected by microdialysis (Yamada et al., 2011). The differences in the amounts of FL-tau and truncated tau between CSF and culture medium could be explained by the three following reasons. First, all of the above observations are based on the use of antibodies to capture tau in the extracellular space. Because of this, one has to take into consideration that, depending on tau conformation and aggregation state in CSF and culture medium, the antibodies might have differential access to their epitopes. Consequently, this could affect the quantitation of FL-tau and cleaved tau. Secondly, the age and conditions of cultures could influence tau species released by neurons. For example, young cultures might release more FL-tau than old cultures, which might contain proteases able to cleave tau that would have been activated following an accumulation of insults. Thirdly, the turnover of extracellular FL-tau might differ in culture medium and CSF. For example, it might be higher in the CSF than in the culture medium. In such a case, a lower amount of FL-tau would be found in the CSF than in the culture medium. Phosphorylation of tau was also examined in both CSF and culture medium. The increase of phosphorylated tau in CSF is extensively used as a biomarker for AD (Hampel et al., 2010). However, in culture medium, most studies reported that tau presents either no phosphorylation or low phosphorylation levels (Mohamed et al., 2014; Rodriguez et al., 2017; Wang et al., 2017). More recently, two studies reported that an important amount of tau in the CSF of AD patients is not phosphorylated (Lewczuk et al., 2017; Ermann et al., 2018). In the case of tau aggregation, most studies did not report the presence of aggregated tau in culture medium and CSF. This could be explained by the lack of sensitivity of the techniques that were used. Indeed, tau aggregates were recently detected in both culture medium and CSF using a seeding assay (Takeda et al., 2016; Merezhko et al., 2018).

The PTMs of exosomal tau were also examined. Most studies reported that both FL-tau and C- and N-terminal truncated tau was found in exosomes, truncated forms being more abundant than FL-tau as noted for free-tau (Saman et al., 2012; Simon et al., 2012; Dujardin et al., 2014; Guix et al., 2018). In some studies, exosomal tau was found to be oligomeric or aggregated (Saman et al., 2012; Asai et al., 2015; Wang et al., 2017). Discrepancies are found in the literature concerning the phosphorylation levels of exosomal tau. Some studies reported that it was phosphorylated whereas in other studies, low phosphorylation levels were observed (Saman et al., 2012; Wang et al., 2017).

In adult human brain, six tau isoforms (0N3R, 1N3R, 2N3R, 0N4R, 1N4R, and 2N4R) are expressed (Buée et al., 2000). Very sparse information exists on the amount of each of these isoforms in the CSF and culture medium in both physiological and pathological conditions. In control CSF, the isoform 1N/3R was reported to be the most abundant (Barthelemy et al., 2016). A decrease of the 4R isoforms was observed in AD and other tauopathies such as progressive supranuclear palsy (PSP) and corticobasal degeneration (CBD), compared to controls (Luk et al., 2012). The preferential aggregation of the 4R isoforms in PSP and CBD could explain the decrease of these isoforms in the CSF (Lee et al., 2001). In the case of AD, where the 3R and 4R isoforms aggregate, the selective decrease of 4R remains unexplained.

Altogether, the above data reveal that several forms of tau co-exist in both the culture medium and CSF and strongly suggest that CSF-tau corresponds to secreted tau.

## How the Elucidation of Tau Secretory Pathways and the Characterization of Extracellular Tau Can Help to Elaborate Therapeutic Strategies for Preventing the Propagation of Tau Pathology

### Elucidation of Tau Secretory Pathways in Physiological Conditions: Understanding the Normal Function of Extracellular Tau

The fact that tau is released by neurons in physiological conditions strongly suggests that it has a role in the extracellular space. This was demonstrated for other cytosolic proteins. For example, annexin A4 and A5, calcium and phospholipids-binding proteins, act as anticoagulant factors via their Ca2+-regulated binding to phospholipids in the extracellular space (Gerke and Moss, 2002). Heat shock proteins are also secreted in the extracellular space (Santos et al., 2017). It was suggested that these proteins can trigger immunosuppression when they are released in the extracellular space under physiological conditions (Tanaka et al., 2007). No study has examined the normal function of extracellular tau. Previous studies indicate that tau could act as a signaling protein by binding to receptors such as muscarinic receptors. Indeed, extracellular tau was shown to bind and activate the M1 and M3 muscarinic receptors (Gomez-Ramos et al., 2008, 2009). However, it remains to be demonstrated whether the concentration of extracellular tau in physiological conditions could exert such effects.

Unconventional secretion of cytosolic proteins could also take place to prevent PTMs that can inactivate them. For example, the unconventional secretion of FGF2 prevents its O-glycosylation that renders its biologically inactive (Wegehingel et al., 2008). The PTMs of extracellular tau are still poorly characterized. It will be important to compare the PTMs of extracellular and intracellular tau to better understand the function of each of these pools. Unconventional secretion of protein could also aim at preventing inappropriate interactions. All these possibilities remain to be explored for tau. A better understanding of the distinct function of each extracellular tau form is crucial when designing anti-tau antibodies for immunotherapy as the sequestration of tau species having a physiological role by an antibody could accelerate neurodegeneration or be associated with significant side effects.

### Elucidation of Tau Secretory Pathways in Pathological Conditions: Preventing and/or Enhancing the Release of Pathological Tau

**FIGURE 2:**
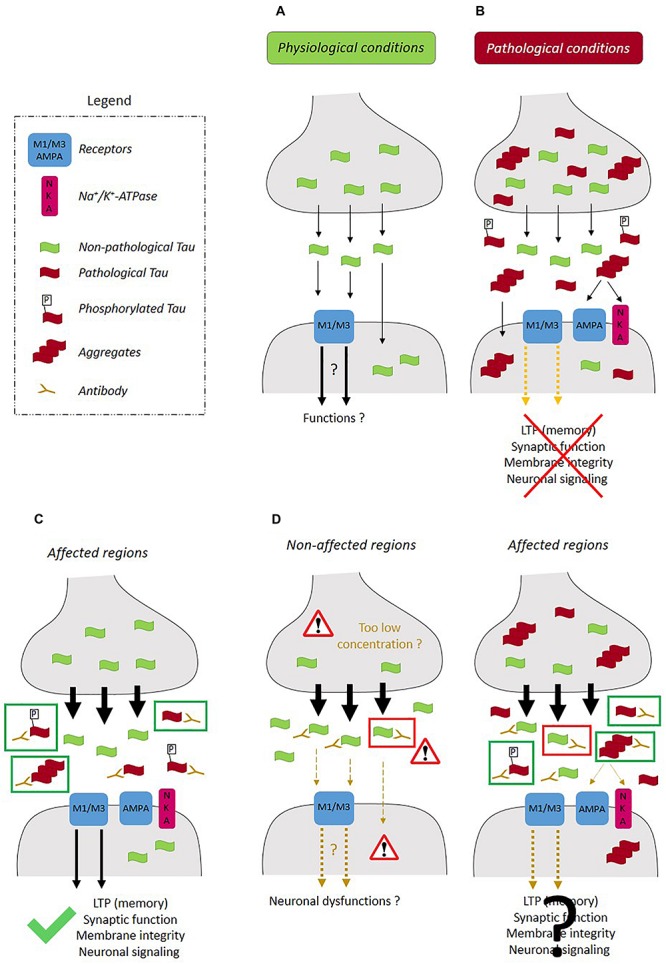
Therapeutic strategies for prevention of the propagation of tau pathology. **(A)** In physiological conditions, tau released at the pre-synaptic terminal presenting low phosphorylation levels, can interact with the receptors such as M1/M3 muscarinic receptors and activate signaling pathways involved in neuronal function. **(B)** In pathological conditions, extracellular pathological tau (phosphorylated and oligomeric) can interfere with synaptic function by inducing deficits in LTP, deficits in K+-evoked glutamate release, alterations in local Ca^2+^ dynamics and by altering the synaptic composition of NKA and AMPA receptors. The most **(C)** efficient therapeutic strategy would prevent the intracellular accumulation and aggregation of pathological tau by increasing its secretion (black arrows) and not that of physiological tau and would also involve the sequestration of pathological tau by an anti-tau antibody in the extracellular space (green rectangle). **(D)** Most pathways involved in tau secretion contribute to the release of tau in both physiological and pathological conditions. In such a case, the activation of tau secretion would not be confined to the affected regions and could also take place in non-affected regions leading to a decrease of intracellular physiological tau and an increase of extracellular physiological tau, which could be detrimental to healthy neurons. Furthermore, the capture of extracellular physiological tau by an anti-tau antibody could be detrimental in non-affected regions (red rectangle). In affected regions, both physiological (red rectangle) and pathological tau (green rectangle) could be released and captured by the anti-tau antibodies. If extracellular physiological tau plays a role in synaptic function and neuronal signaling, its capture could compromise neuronal recovery in the affected brain regions.

Several observations indicate that blocking tau secretion could be beneficial by preventing the propagation of tau pathology in the brain and the detrimental effects of extracellular tau on synaptic function. Tau pathology is believed to propagate *trans-*synaptically by the release of tau at the pre-synaptic terminal and its uptake by the post-synaptic neurons (Calafate et al., 2015; Goedert and Spillantini, 2017). This cascade of events was confirmed in a recent study (Katsinelos et al., 2018). When tau translocation across the PM was blocked in pre-synaptic neurons, transcellular spreading of tau to post-synaptic neurons was prevented. Extracellular tau could also exert detrimental effects on synaptic function (Figures 2A,B). Extracellular tau secreted from human-derived iPSCs differentiated neurons induced deficits in long-term potentiation (LTP), a synaptic event underlying memory, when it was injected in mouse brain (Hu et al., 2018). It was also reported that a soluble and unaggregated N-terminal fragment shown to accumulate in the CSF of AD patients induced a pre-synaptic deficit in K+-evoked glutamate release on hippocampal synaptosomes along with an alteration in local Ca^2+^ dynamics (Florenzano et al., 2017). Furthermore, prolonged exposure of this peptide induces neuritic dystrophy, microtubule breakdown, deregulation in pre-synaptic proteins and loss of mitochondria located at the nerve endings. In a recent study, tau fibrils composed of either 1N3R or 1N4R isoform were shown to form clusters on the PM of excitatory synapses (Shrivastava et al., 2019). This clustering of tau fibrils was mediated by their interaction with Na+/K+-ATPse (NKA) α3 subunit and the AMPA receptor subunit GLUA2. Upon tau-fibril clustering, a decrease of α3-NKA was observed resulting in a decrease capacity of restoring Na+ level after depolarization. An increase of GLUA2-AMPA was also observed, which induced higher Ca2+-influx following action potential. A higher activity of M1 and M3 muscarinic receptors induced by extracellular tau could also contribute to synaptic dysfunction. Interestingly, non-phosphorylated tau abundant in the extracellular space but not phosphorylated tau was shown to interact with these receptors (Gomez-Ramos et al., 2006, 2008; Diaz-Hernandez et al., 2010). Lastly, extracellular tau could affect synapses by altering the biophysical properties of lipid rafts (Flach et al., 2012).

Conditions of stress might harm the degradative pathways such as proteasome and/or autophagy normally used to eliminate pathological tau. In such conditions, its secretion could be a very efficient clearance mechanism to prevent its intracellular accumulation and aggregation. This was recently demonstrated for TDP-43, a protein linked to ALS (Iguchi et al., 2016). Blocking TDP-43 secretion increased significantly its aggregation and toxicity. It is still unclear if preventing tau secretion will be detrimental to neurons.

Interestingly, the time course of the propagation of tau pathology in the brain and the amount of tau in the CSF differ among tauopathies. In AD where the progression of the disease is slow, an increase of tau in the CSF is observed whereas in other tauopathies such as PSP and CBD, where the disease progression is fast, no change or a small decrease of tau is noted in the CSF (Lee et al., 2001; Jabbari et al., 2017). At first glance, these observations do not support that extracellular tau is involved in the propagation of tau pathology. In a recent study, PSP-tau and CBD-tau species were reported to be more potent than AD-tau species at inducing propagation, meaning that they induced it faster and earlier than AD-tau (Narasimhan et al., 2017). Three explanations can reconcile all of the above observations. Firstly, in PSP and CBD, it is possible that tau secretion is lower than in AD but the tau species that are released are more potent than AD-tau at inducing the propagation of tau pathology. Lower secretion levels of tau would fit with the fact that PSP- and CBD-tau have a higher capacity to aggregate than AD-tau. Indeed, in transgenic mice, a decrease of tau in the CSF was correlated to the increase of tau aggregation (Yamada et al., 2011). Secondly, the secretion levels of tau are similar in PSP, CBD and AD but the uptake of PSP- and CBD-tau species is increased when compared to AD-tau resulting in a lower CSF concentration but faster propagation than AD-tau. Thirdly, in both PSP and CBD, tau pathology is noted in glial cells and neurons whereas in AD, tau pathology is mainly neuronal (Lee et al., 2001). Tau released by glial cells might be less abundant but more potent to induce propagation in both neurons and glial cells. Microglia can facilitate tau pathology propagation between neurons by phagocytosing and exocytosing tau protein (Guix et al., 2018). In the case of astrocytes, it was reported that they could decrease the spreading of tau pathology by taken up extracellular tau and targeting it for degradation (Martini-Stoica et al., 2018). However, in PSP and CBD, the tau taken up by astrocytes might be exocytosed instead of degraded.

Based on the above observations, one could speculate that blocking tau secretion could prevent the propagation of tau pathology and the detrimental effects exerted by extracellular tau on synaptic function. On the other hand, this could accelerate the intracellular accumulation and aggregation of pathological tau in the somato-dendritic (SD) compartment as noted for TDP-43. In either case, the elucidation of tau secretory pathways is central for the elaboration of a therapeutic strategy to prevent the development of tau pathology and its propagation. An efficient therapeutic strategy should prevent the intracellular and extracellular accumulation of pathological tau. To achieve this, an increase of pathological tau secretion for prevention of its intracellular accumulation and its sequestration in the extracellular space by an antibody to prevent its propagation and synaptic detrimental effects are necessary (Figure 2C). Furthermore, it is important to take into consideration that most of the mechanisms involved in tau secretion are active in both physiological and pathological conditions indicating that blocking them could have detrimental effects in the areas of the brain not affected by tau pathology where extracellular tau could play a role in neuronal function (Figure 2D). An aspect of tau secretion that was neglected in most studies is the fact that it reaches a plateau. This was only reported by Bright et al. (2015). This phenomenon was observed with all the cell types that we used to examine tau secretory pathways (unpublished data). This observation indicates that a feedback mechanism regulates tau secretion. Taking this into account, the administration of an anti-tau antibody that would block this mechanism by binding to the region of tau involved in it, could lead to an increase of tau secretion. In such a case, the sequestration of pathological tau by the antibody would be limited because of the sustained release of tau compromising the beneficial effects of this antibody. All the above concerns and questions will need to be addressed before the elaboration of therapeutic strategies based on tau secretion. Furthermore, the identification of tau secretory pathways will help to determine whether other proteins are secreted by these pathways, which could contribute to neuronal dysfunction and/or could be used as novel biomarkers for tauopathies.

From recent studies, one could conclude that combined therapeutic strategies with tau secretion would be the most efficient way for preventing the propagation of tau pathology in the brain. Three combined strategies could be elaborated. Firstly, a recent study reported that impaired dural lymphatic function resulted in an increased retention of extracellular tau in the brain as well as a delayed clearance of tau to the periphery (Patel et al., 2019). A therapeutic strategy combining an increase of tau secretion and an increase of tau clearance by the dural lymphatic system could be very efficient in preventing intracellular and extracellular accumulation of tau in the brain. Secondly, intraneuronal transfer of tau was shown to occur by the formation of tunneling nanotubes (Tardivel et al., 2016). This implies that both tau secretion and formation of tunneling nanotubes would have to be blocked to prevent tau pathology propagation. Thirdly, extracellular tau can be taken up by astrocytes. Such an uptake was shown to reduce tau spreading (Martini-Stoica et al., 2018). A concomitant increase of tau secretion and tau uptake by astrocytes could prevent extracellular and intracellular tau accumulation.

Lastly, a recent study reported that END-binding proteins found at the tip of microtubules could modulate tau secretion in non-neuronal cells indicating that microtubules could play a determinant role in the release of tau by neurons (Sayas et al., 2019). Although this remains to be demonstrated in neurons, microtubule network could be modulated to increase tau secretion, which could be combined with the administration of an anti-tau antibody for sequestration of secreted tau.

### Characterization of Extracellular Tau Species in Physiological and Pathological Conditions: An Essential Step for Improving the Efficacy of Tau Immunotherapy and Improving the Use of Tau as a Biomarker

The non-vesicular extracellular tau, the main target for immunotherapy, was shown to be able to transfer from neuron to neuron (Takeda et al., 2015; Katsinelos et al., 2018). Several studies have reported that tau aggregates are necessary to induce propagation. The major drawback of these studies is that aggregates composed of recombinant tau, which does not present the PTMs of secreted tau such as cleavage and phosphorylation, was used instead of tau purified either from culture medium or CSF (Wu et al., 2012; Mirbaha et al., 2015). Tau aggregates isolated from the brain of patients presenting a tauopathy were also used in these studies (Takeda et al., 2015; Narasimhan et al., 2017). However, it is not clear if these aggregates are released and/or formed in the extracellular space in AD brains. In recent studies where new sensitive techniques were used, aggregates of tau were detected in the culture medium and CSF indicating that if aggregates are needed to induce the propagation of tau pathology in the brain, extracellular tau could do it (Takeda et al., 2016; Merezhko et al., 2018). In the case of phosphorylation, it is less clear if it is necessary for inducing propagation. Most studies reported that extracellular tau presents low levels of phosphorylation, an observation recently confirmed in human CSF (Le et al., 2012; Mohamed et al., 2014; Lewczuk et al., 2017; Rodriguez et al., 2017; Wang et al., 2017; Ermann et al., 2018). Aggregates composed of non-phosphorylated recombinant tau were shown to induce tau propagation (Wu et al., 2012; Mirbaha et al., 2015). Cleavage of extracellular tau seems to increase its spreading and toxicity. In lamprey, the N-terminal domain of tau was sufficient for secretion and propagation (Le et al., 2012). However, the pattern of secretion differed between the N-terminal and FL-tau in this system. The N-terminal fragment presented a diffuse secretion occurring at the somata whereas FL- tau presented a focal secretion taking place in dendrites and the axon. Interestingly, extracellular N-terminal fragments of tau were shown to increase the production of Aβ in primary human cortical neurons (Bright et al., 2015). When these fragments were neutralized by an antibody, Aβ production was decreased.

The isoform composition of extracellular tau remains to be well characterized for most tauopathies. This point is important since it was recently reported that tau isoforms do not have the same propensity for spreading in the brain. Indeed, the 4R isoforms were found to propagate farther than 3R tau (Dujardin et al., 2018). In the same study, it was reported that misfolded tau composed of either P301L or P332S tau mutant linked to FTLD-tau presented a spreading more distant from the initiation site than hyperphosphorylated tau whereas the opposite was noted for wild-type tau (Dujardin et al., 2018). Based on these observations, antibodies targeting hyperphosphorylated tau might not be as efficient as antibodies targeting misfolded tau in blocking tau propagation in patients presenting these mutations. Altogether, the above observations indicate that extracellular tau could induce the propagation of tau pathology in the brain. However, a better characterization of secreted tau PTMs and isoforms is needed for a more complete understanding of its function and detrimental effects in the extracellular space. Most importantly, the analysis of tau PTMs and isoforms in physiological and pathological conditions will help to design antibodies that will sequester the pathological not the physiological species.

It remains to be demonstrated whether CSF-tau could be used for the differential diagnosis of tauopathies at early stages of the disease and/or for following the progression of a tauopathy. The amount of total and phosphorylated tau is increased in the CSF of AD patients (Hampel et al., 2010). More recently, it was demonstrated that non-phosphorylated tau was also increased in the CSF of these patients (Lewczuk et al., 2017). The use of CSF-tau as a biomarker for tauopathies other than AD such as PSP is less clear. Some studies reported an increase of CSF-tau in PSP whereas others noted either no change or a decrease (Jabbari et al., 2017). In the PSP studies, the analysis of CSF-tau was performed with the anti-tau antibodies used for AD. The lower detection of tau in PSP than in AD could indicate that extracellular tau in PSP and AD differs and therefore that antibodies used in AD may not be able to detect tau in PSP. A better characterization of extracellular tau in PSP and AD is needed to demonstrate this. Another explanation could be that tau is less secreted in PSP, leading to a faster accumulation and aggregation of intracellular pathological tau, which would explain the faster progression of the disease compared to AD. A better characterization of tau secretion pathways will help to demonstrate whether these pathways are less active in PSP.

## Conclusion

In conclusion, the elucidation of tau secretory pathways is central to further investigate the physiological function of extracellular tau and its contribution to tau pathology. Extracellular tau is the main target of therapies involving the administration of an anti-tau antibody. A better characterization of all tau forms released in the extracellular space and their function is needed for the design of more efficient antibodies that will solely sequester toxic tau species. This characterization will require the use of more sensitive techniques for the detection of extracellular tau PTMs and aggregation state in the CSF.

## Author Contributions

CP, AD, and NL wrote the manuscript. CP prepared the figures.

## Conflict of Interest Statement

The authors declare that the research was conducted in the absence of any commercial or financial relationships that could be construed as a potential conflict of interest.
